# Responding to Domestic Violence in General Practice: A Qualitative Study on Perceptions and Experiences

**DOI:** 10.1155/2012/960523

**Published:** 2012-11-06

**Authors:** Howa Yeung, Nubaha Chowdhury, Alice Malpass, Gene S. Feder

**Affiliations:** ^1^New York University School of Medicine, New York, NY 10016, USA; ^2^Department of Emergency Medicine, Maimonides Medical Center, Brooklyn, NY 11219, USA; ^3^Centre for Academic Primary Care, NIHR National School for Primary Care Research, School of Social and Community Medicine, University of Bristol, Bristol BS8 1TH, UK

## Abstract

The perceptions and experiences among general practitioners (GPs) and nurses in identifying female patients experiencing domestic violence and referring patients to specialist agencies need to be clarified. Eleven GPs and six nurses participating in a multidisciplinary domestic violence training and support programme in east London and Bristol were interviewed. All participants recognised that identification of women experiencing domestic violence and offering support were part of their clinical roles. Perceived differences between GPs and nurses, including time constraints, level of patient interaction, awareness of patients' social history, scope of clinical interview, and patient expectations were used to explain their levels of domestic violence inquiry. Barriers to inquiry included lack of time, experience, awareness of community resources, and availability of effective interventions postdisclosure. Longstanding relationships with patients were cited both as barrier and facilitator to domestic violence disclosure. Some nurses reported discomfort with direct inquiry due to the lack of clinical experience in responding to domestic violence despite satisfaction with training. Future domestic violence training programmes should take into account potential differences between GPs and nurses, in terms of their clinical roles and the unique barriers encountered, in order to improve self-efficacy and to facilitate collaborative and effective responses.

## 1. Introduction

Domestic violence is a pattern of threatening behaviour, violence, or abuse including psychological, physical, sexual, financial, or emotional abuse between adults in the same family or who are or have been intimate partners [1]. It is a severe breach of human rights with profound consequences, particularly for women who, compared to men, experience more sexual violence, more severe physical violence, and more coercive control from their partners [2, 3]. Domestic violence threatens women's physical health, mental health, and social functioning, and poses a serious public health problem [4, 5]. Its impact is widespread internationally, with lifetime prevalence ranging from 15% to 71% in a WHO multicountry study [6]. In the UK, 30% of women have experienced domestic abuse in their lifetime and 7% within the past year [7]. Its prevalence in women seeking healthcare is even higher; among women attending general practices in east London, 41% had experienced physical or sexual violence in their lifetime and 17% within the past year [8].

Primary care clinicians potentially have a key role in the identification of and initial professional response to domestic violence [9]. Of note, abused women are more likely to be in touch with health services than any other professional agencies [10, 11]. Since abused women frequently seek health care before disclosure, clinical settings could provide a safe and nonjudgmental context for victims to discuss what they are experiencing [11–14].

Despite their potentially important role, most doctors and nurses have received little or no training and are currently ill prepared to provide an effective response to domestic violence by often failing to identify and document it [4, 8, 10, 15, 16]. When women do disclose abuse to clinicians, there is evidence of inappropriate and poor-quality responses [11, 16]. System-level interventions, in particular training and referral pathways, aim to increase disclosure and promote referral to domestic violence services, but to date have yielded inconsistent results, with a lack of evidence showing improvements in health care response to domestic violence [17, 18].

Studies on the knowledge, attitudes, and beliefs of physicians and nurses in domestic violence response have revealed important barriers to addressing domestic violence [19, 20]. Commonly cited barriers include the lack of knowledge, training, patient disclosure, self-efficacy, system-level support, and time [19, 20]. Effective identification of women experiencing violence needs to be a key component of training since it is challenging even for those physicians committed to helping survivors [21]. We know from a systematic review of qualitative studies that survivors of domestic violence want to be asked by doctors but not pressured to disclosure, but patient disclosure is a prerequisite for clinician engagement with domestic violence [9, 11]. Most qualitative research on domestic violence competencies and training needs of clinicians in primary care has focused on doctors and nurses separately. There is little research distinguishing potentially different professional roles of physicians and nurses that may influence their responses to domestic violence in general practice. The objectives of this study were to explore the perceptions and experiences of general practitioners (GPs) and practice nurses on addressing domestic violence before and after participation in a domestic violence training and support programme.

## 2. Methods

This qualitative study was nested in the identification and referral to improve safety (IRIS) trial, the first European pragmatic clustered randomised controlled trial to assess the effectiveness of a domestic violence training and support programme targeting general practices. The IRIS intervention was centred on multidisciplinary training sessions for general practitioners and nurses addressing inquiry about abuse and appropriate responses to disclosure, coupled with a referral pathway to specialist domestic violence advocacy and periodic reinforcement and feedback from domestic violence advocate-educators [22]. The programme was designed to improve recording and management of domestic violence and aimed to increase rates of domestic violence identification and referral to advocacy agencies. The main findings of the trial showed a 3-fold difference in identification rates and a 22-fold difference in recordings of referrals between the 24 intervention and 24 control practices [23]. An economic analysis of the trial showed that the programme is likely cost-effective and potentially cost-saving [24].

In our study, two phases of interviews were conducted from July to August 2007 and July to August 2008. Clinicians from the 24 general practices participating in the intervention arm of the IRIS trial in Hackney and Bristol were approached for interviews. 

### 2.1. Recruitment and Interview Procedures

Semistructured interviews lasting 30 to 60 minutes were conducted by two researchers (NC and HY). Both were medical students from the United States and external to the IRIS trial who had completed coursework in qualitative methods and received independent funding to conduct this research. Phase I interviews were conducted by NC with a convenience sample of general practitioners and nurses in Hackney, east London prior to the IRIS intervention. Phase II interviews were conducted by HY after two IRIS training sessions were completed. During phase II, follow-up interviews with clinicians in the same Hackney practices from phase I were attempted. GPs and nurses from Bristol were also contacted for interviews also in phase II. Recruitment of the Bristol sample was purposive, emphasising practices with low referral rates and taking into account clinician type, gender, practice size and socioeconomic status of the practice catchment area to maximise diversity of responses. All sampled clinicians were initially contacted by e-mail and followed up with telephone calls. If the clinician could not be contacted or declined to participate, another clinician from the same practice or level of referral was contacted.

Clinician demographics, including age, sex, and practice experience, were collected by direct inquiry before the interviews began. Topic guides covering clinician knowledge, beliefs and practices on domestic violence cases, barriers and facilitators in inquiry and referral, and expectations of domestic violence training programme were used in each phase of interviewing. Programme evaluation questions on the IRIS intervention were added to the topic guide in the phase II. The topic guides in each phase were revised after two pilot interviews and then used for the remainder interviews (see the appendix). The interviews were recorded and transcribed verbatim.

### 2.2. Data Analysis

Interview data were coded and analysed by the framework approach [25]. Themes were articulated from coding a sample of transcripts with additional *a priori* themes from the topic guide on barriers and facilitators to domestic violence inquiry and response. The data from both phases were independently coded by NC and HY, and then grouped and rearranged into a chart of key abstracted themes. Common and divergent themes between pre- and postintervention interviews and between doctors and nurses were noted. Differences in coding between NC and HY were resolved by discussion and input from AM and GF.

## 3. Results

### 3.1. Participants

Seventeen clinicians—11 general practitioners and 6 nurses—participated in our study. In phase I, three doctors and two nurses from Hackney practices participated. In Phase II, follow-up interviews were conducted with one doctor and two nurses from the same practices in Hackney. In addition, seven doctors and two nurses from Bristol participated in phase II. 

Five of 11 general practitioners and all six nurses interviewed were female. The doctors reported working as clinicians on average for 10.2 years in their current practice, and 18.8 years in total. The nurses reported working as clinicians on average for 9.3 years in their current practice, and 12.2 years in total. Six out of 12 clinicians interviewed in phase II were recruited from practices with low levels of referral in the IRIS trial.

### 3.2. Role in Addressing Domestic Violence

All of the informants, both before and after training, agreed that they have a role in addressing domestic violence as a health care professional.

*“We are in the ideal position to find out about it. I think that there has to be a combination of people being encouraged to disclose… and then we should be available as we are for anything to hear about it… It is easier because we have permission to ask questions without being criticized… That's the nature of our job. And we're in a very privileged position.” (GP 1, Phase I, Hackney)*

The roles elicited in the interviews included: being aware of the issue, identifying domestic violence cases, encouraging disclosure and action, providing information and support, treating associated medical conditions, assessing safety risks, referring to external agencies, and following up on patient status. Many clinicians highlighted the focused nature of their roles, mainly in identifying cases and providing help to patients through referral to external agencies, as opposed to offering intensive counselling themselves.

*“I have sort of a low-grade counselling skill that I will use on everyone. I don't want to become anything more than that because… we deal with everything in primary care… I prefer to refer them on… but I want to support them in their intermediate phase.” (GP 1, Phase I, Hackney)*

*“I don't need to know how to deal with the whole situation, how to get them out, how to deal with all such things, ‘cause that isn't part of my role. It's my role to refer and to follow up, like to see your patients again and check everything is okay.” (Nurse 2, Phase II, Bristol)*

### 3.3. Perceived Differences between General Practitioners and Nurses

Both doctors and nurses perceived differences in their roles on domestic violence. Aspects of nursing within general practice that were perceived as advantages in comparison to doctors included: longer consultations, more interactions with patients, and potential greater awareness of patients' social history.

*“With the nurses, I guess they live in the area, but I think they spend longer with the patients. They know some of the patients. They might even know the history to patients' relationships… so they might just have a little bit of awareness, perhaps not make it so easy for the patients to open up, but at least for them to sort of advise the patients perhaps they could open up to somebody if they were interested in it.” (GP 6, Phase II, Bristol)*

Perceived patient attitudes that inhibit disclosure to doctors, which may not necessarily be present with nurses, were also noted as a barrier to disclosure.

*“You feel sometimes you've probed far enough, because people may have domestic violence, but they are not going to tell me, because you're the doctor. They have to tell someone else.” (GP 3, Phase II, Bristol)*

*“I think that a lot of people think that they would best not bother a doctor, whereas a nurse they seem as more personable and more approachable potentially… I don't know, probably an old-fashioned stigma between doctors and nurses, and nurses are more respected in that sense.” (Nurse 2, Phase II, Bristol)*

On the other hand, nurse consultations are more task-oriented than GP consultations, often focusing on reviews for chronic conditions, potentially limiting the opportunity to probe about domestic violence.

*“Perhaps as a nurse, ‘cause you have a different role from the doctors, it might not come up in the same way… I might tend to see people, for example, who perhaps someone with bruises… and then I'd ask. But I suppose when you consider the nature of the consultation, I haven't [these] patients… From what I understand, the doctors here they see it or they address it much more often… so perhaps they learned their way and how to deal with it.” (Nurse 1, Phase II, Hackney)*

With regards to referring identified domestic violence cases to the specialist of domestic violence, both nurses interviewed in phase I would defer referrals until after consultations with the doctors.

*“We work very closely with the GPs and I think if I discovered anything or was told anything, I would first want to discuss it with the GP concerned, particularly because the GPs here are very sort of hands-on and I'm not too sure, I think they'd want to be involved if you were to go off to talk to other agencies.” (Nurse 1, Phase I, Hackney)*

While both general practitioners and nurses were trained to refer patients directly to the specialist agencies in the IRIS intervention, only one nurse interviewed in phase II said that she would directly refer the patient herself while the other nurse informants said they would consult with the doctors before referral.

*“I would probably do a direct referral, but I would mention it to the GP, so that the GP is aware of what's going on. But I wouldn't need the GPs to refer.” (Nurse 2, Phase II, Bristol)*

“*Here we have this very much this sort of thing about liaising with… Because I don't feel that I know the patient best, because I might see them once a year, but the doctors are seeing them on a fairly regular basis. So I mean I'll feel happy discussing the issues with the doctor.” (Nurse 2, Phase II, Hackney)*

### 3.4. Barriers and Facilitators

Both patient and provider barriers were reported by doctors and nurses. Cited patient barriers included embarrassment, confidentiality issues, normalisation, fear of consequences, presence of other family members in the clinic, and cultural barriers. Elicited provider barriers included time constraints, lack of experience in responding to domestic violence, patient reluctance to disclosure, lack of awareness and mistrust in community resources, lack of effective interventions, and stereotypes toward patients facing domestic violence.

The quality of the clinician-patient relationship acted both as a barrier and a facilitator. Longstanding patient relationships were cited by some clinicians as deterring clinician inquiry and patient disclosure. Social stigma associated with domestic violence may hinder disclosure in the context of longstanding patient relationships. This was the case for both GPs and nurses.

*“You have people who… you know very well, you know who their partners are, you see them in the practice… it may not even occur to you that person could be violent, so that's probably why you may not [ask]—I may not so much for somebody I know well.” (GP 1, Phase II, Hackney)*

*“A lot of people I think don't necessarily want their GP to know [about domestic violence], ‘cause their GP knows the whole of the family… [the patient] might know their GP even socially, some patients do, so therefore they don't really want to bother them with this kind of stuff… they wouldn't necessarily want to tell them that you've actually got problems in this “perfect family” that you've been presenting with.” (Nurse 2, Phase II, Bristol)*

On the other hand, some clinicians also noted that close patient relationships could be facilitative. They could utilise the trust built in the relationship to facilitate deeper discussion of home life, potentially leading to disclosure and action.

*“I think it's about trying to facilitate discussion and establish trust, and I think it's such a big thing for people to share with you… you want to aid people in facilitation so it's probably the way you consult on it” (GP 1, Phase II, Hackney)*

### 3.5. Inquiring about Domestic Violence and Adequacy of Training

Most general practitioners from phases I and II expressed confidence in directly inquiring about domestic violence, especially when signs and symptoms are apparent. However, it was unclear how often they asked directly about domestic violence as many preferred indirect routes of inquiry, which allowed for more exploration around home issues before questioning about domestic violence.

*“It doesn't bother me to ask them… But I think it's sometimes better to ask them in a roundabout to explore around it. And if you get a few hints, then you can ease into the direct statement.” (GP 1, Phase I, Hackney)*

*“We might not pick it up… we may not think to ask always when we should perhaps, but I think if we think it's an issue, I don't as a doctor here would have any reluctance in asking.” (GP 1, Phase II, Hackney)*

In contrast, nurses' comfort levels about direct inquiry ranged widely depending on their level of clinical experience addressing domestic violence. Two nurses who expressed relative ease with direct inquiry cited their backgrounds working in Accident & Emergency department and in Women's Aid. Three nurses who expressed discomfort cited their lack of experience in directly engaging patients experiencing domestic violence, even if they had training in domestic violence response.

Most clinicians interviewed after the IRIS intervention expressed satisfaction with the training in raising the profile of domestic violence issues in their practice and in promoting awareness of community referral resources. Major concerns about the programme included the large amount of time devoted to training and long-term maintenance of training effects. Despite the success of the training programme in promoting awareness, some nurses expressed the need for practical experience in order to gain confidence in addressing domestic violence.

*“I don't necessarily think they haven't trained me adequately… I think it's a practical thing about, if you've experienced it more, if you've seen more, and you've had more practice at addressing the patient or whatever, you'd become more confident in it. Not necessarily saying that they haven't trained—given enough training— ‘cause I think I'm quite aware. A lot of it is about practice.” (Nurse 1, Phase II, Hackney)*

## 4. Discussion

Within the context of a system-level training and support intervention, this study detailed the perceptions and experiences of general practitioners and nurses in addressing domestic violence. It showed that the clinicians all recognised their roles in addressing domestic violence, which is consistent with the results of our baseline survey of practices participating in the IRIS trial [26] and with a previous survey showing that most health professionals believe domestic violence is a healthcare issue [15, 26]. The major perceived provider barriers perceived by our informants, including the lack of time and experience, stigma attached to patient disclosure, and lack of effective postdisclosure interventions, are also consistent with those found in previous studies on domestic violence screening and management by healthcare providers [16, 19, 20]. These common barriers across studies highlight the challenges in developing an effective domestic violence response in general practices that have yet to be overcome.

This study adds to the current literature by identifying new system-based factors that may shape domestic violence identification and referral patterns [23, 27]. Both GPs and nurses described the limitations of their own professional roles as barriers to disclosure, each viewing the other as better placed to ask women about domestic violence. We identified two forms of deference as barriers to disclosure: doctors' perceptions of patient deference in “*best not bother a doctor*” in their home lives and nurses' professional deference toward their physician colleagues to refer patients externally. This second form of deference may reflect a convention within primary care: a preference for nurses to refer patients to GPs within the practice instead of external agencies. Indeed, a similar pattern was seen in the response to mental health problems in UK general practices, for which 95% of practice nurses referred the patient to GPs while only 22% liaised with community mental health professionals. A recent Swedish study on primary care nurses' response to domestic violence also showed that their most common intervention was to refer the patient to a doctor [28]. Therefore, different perceptions of the roles of GPs and nurses in identifying and referring patients experiencing domestic violence might in part explain the preponderance of referrals from GPs over nurses in the IRIS trial despite joint training of both groups of clinicians in direct referral to external agencies [23, 27].

Our study highlights the need to further elucidate professional differences in attitudes and practices toward addressing domestic violence among members of general practices. Within the IRIS cohort of general practices, GPs were overall better prepared and more knowledgeable than practice nurses in addressing domestic violence prior to training [27]; the opposite was shown in a previous survey study on various healthcare workers [29]. One previous survey reported that practice nurses were significantly less likely than GPs to think domestic violence screening should take place [15]. In our study, some nurses reported feeling less prepared due to a lack of clinical exposure to responding to domestic violence cases. Their anecdotal responses are consistent with a randomised controlled trial on domestic violence screening, showing that more women reported contacts with family physicians for violence-related services than with nurses or nurse practitioners [30].

Of note, nurses who cited inadequate self-efficacy in domestic violence response in our study attributed it to the lack of professional experience rather than the lack of adequate training. These results are consistent with a Canadian survey of physicians and nurses which found professional experiences to be as important as formal training in increasing perceptions of preparedness, self-confidence, comfort initiating domestic violence discussions, and comfort with discussions following disclosures [19]. Interestingly, that study also showed that among clinicians with no previous domestic violence education, nurses felt less prepared in addressing the issue—a finding that disappeared when training is present [19]. These results suggest a need to tailor future interventions to account for the different clinical roles, unique barriers, and professional experience with addressing domestic violence among GPs and nurses, in order to improve clinician self-efficacy and to provide appropriate responses to patients experiencing domestic violence.

This study should be reviewed in the context of its limitations. First, informants were based in general practices participating in a randomised controlled trial. Thus, our findings should not be generalised to all clinicians, who conceivably may have lower engagement with the issue of domestic violence. Purposive oversampling of clinicians from low-engagement practices in phase II may have mitigated part of this sampling bias and provided a wider range of responses. Second, the phase I informants were based on a convenience, rather than purposive or random sample. Third, we recognise that thematic saturation was not achieved in some themes due to the small sample size and there may exist a wider range of perceptions within those themes we detected. Fourth, the small number of follow-up interviews (i.e., phase I informants interviewed in phase II) allowed few comparisons of clinician perceptions before and after the intervention. Thus we are unable to identify the source of differences between doctors and nurses, which may result from differential impacts of the intervention versus more inherent differences in their professional roles. Further qualitative research will be needed to confirm our findings in a more representative sample of clinicians, from the patients' points of view, and in other general practice settings in the UK or internationally.

## 5. Conclusions

Domestic violence remains a major public health and clinical problem with a poor health care response. Within the context of a training and support programme for general practices, we elicited clinicians' perceptions of their roles in the identification and management of patients experiencing domestic violence. General practitioners and nurses had different views of their own and, to some extent, each other's roles concerning domestic violence. These differences in perceived roles are potential barriers to addressing domestic violence in a multidisciplinary clinical setting and may explain different referral patterns to specialist agencies. Domestic violence interventions should take into account the clinical roles and unique barriers encountered by general practitioners and nurses to increase clinician self-efficacy and to provide a collaborative and effective response to domestic violence.

## Appendix

### Topic Guides

Knowledge, Belief, and Practice

1. What do you understand by “domestic violence”?
2. What proportion of your patients do you think have experienced domestic violence?
3. What do you see your role as a health care professional in addressing domestic violence? What are the roles of GPs, nurses, or receptionists?
4. Have you had any training in the past on domestic violence? (phase I)
5. How confident are you in addressing domestic violence in your practice?
6. What makes you suspect domestic violence? How do you recognize domestic violence?
7. What do you do after a patient discloses experiences of domestic violence?

Barriers and Facilitators

(8) How comfortable are you with the idea of directly asking your patients about domestic violence?
(9) What challenges do you face in:
  - Asking women about domestic violence?
  - Offering support to women disclosing that they are experiencing domestic violence?
(10) What, if anything, makes it easier for you in:
  - Asking women about domestic violence?
  - Offering support to women disclosing that they are experiencing domestic violence?

Expectations of IRIS (Phase I)

(11) What changes in the practice with regards to domestic violence would be most rewarding for you?
(12) What do you think will be the most worthwhile aspect of the IRIS education and training programme?
(13) What matters most to you about the service you offer to your patients?

Experience in IRIS (Phase II)

(14) What were your expectations from the IRIS intervention? What did you want to gain from the program?
(15) Did the IRIS intervention meet your expectations? Was the experience worthwhile? Why or why not?
(16) Do you feel adequately trained for domestic violence screening and support? Why or why not?
(17) What aspects of the IRIS intervention were helpful (or not helpful) in improving domestic violence response in the primary care setting? why?
(18) If you have referred someone to the domestic violence advocate-educator, what do you think about his/her response?

Future Direction and Closing

(19) How can future domestic violence interventions be improved?
(20) Is there anything else you would like to discuss before we stop for today?
